# In vitro assessment of the roles of initial preparation size and solvent application on apically extruded debris in endodontically treated teeth

**DOI:** 10.34172/joddd.2023.40665

**Published:** 2023-12-30

**Authors:** Esra Yavaş, Aslıhan Yekeler, Serhat İlgen, Emel Uzunoğlu Özyürek

**Affiliations:** Department of Endodontics, Faculty of Dentistry, Hacettepe University, Ankara, Turkey

**Keywords:** Endodontics, Root canal, Retreatment

## Abstract

**Background.:**

This study investigated the effects of solvent use and initial canal enlargement size on apically extruded debris (AED).

**Methods.:**

The palatal roots of 60 upper molars were assigned to two groups based on the initial preparation size: F3 or F4 ProTaper Universal files. The roots were obturated using the single-cone technique. Each group was subdivided based on the retreatment procedures: with or without solvent. After evaporation, the Eppendorf tubes were weighed using an electronic balance. The data were statistically analyzed (*P*=0.05).

**Results.:**

Using a solvent enhanced the amount of AED in samples initially prepared up to F3 or F4 files (*P*<0.05). When the solvent was used, the AED was greater in samples initially prepared up to the F4 file (*P*<0.05); however, when the solvent was omitted, the results were reversed (*P*<0.05).

**Conclusion.:**

Both parameters had a role in AED. Solvents should be used cautiously during root canal retreatment because of the potential for apical extrusion of filling materials. Using files with smaller tapers during initial preparation might be beneficial when considering the amount of AED compared to those with larger tapers.

## Introduction

 Root canal treatment that saves the tooth with high success rates may fail due to inadequate irrigation protocols, insufficient root canal filling, or coronal leakage.^1^ In the case of primary treatment failure, endodontic retreatment of the root canal without surgical intervention should be considered the first treatment option.^2^ Several techniques, including ultrasonics, lasers, hand files, nickel-titanium (NiTi) rotary files, and solvents, have been devised to remove root canal filling materials from the root canal system.^3,4^ Using conventional manual files to remove root canal fillings is time-consuming and has been associated with some complications.^5^ NiTi rotary instruments are commonly used in endodontics due to their benefits, which include flexibility, centering ability, and low transportation risk.^6^ Extrusion of filling materials, tissue debris, microorganisms and their products, dentin chips, and irrigation solutions beyond the apical foramen has been reported during root canal filling removal.^7-11^ This extrusion can cause postoperative inflammation, flare-ups, and long-term failures.^12^ In addition to previous advantages, NiTi file systems are associated with less apical extrusion during root canal filling removal than hand files.^13,14^ Various NiTi rotary files have been developed specifically to remove filling materials from root canal walls, and their effectiveness, cleaning ability, and safety have been evaluated several times.^15,16^

 Gutta-percha can be dissolved and softened using solvents such as chloroform, eucalyptol oil, xylene, halothane, turpentine oil, and pine needle oil; among these solvents, chloroform is the most preferred.^17^ The mechanical removal of the root canal filling materials can be aided by solvents.^7-10^ The use of a solvent reduced the quantity of apically extruded debris (AED)^8,10^ and the time spent compared to the absence of a solvent, but there are conflicting results regarding this aspect in the literature.^9^

 Every mechanical shaping strategy results in material extrusion from the apex, and the quantity of AED can vary depending on the kinematics, cross-section design, taper, and alloy of NiTi files.^3,4,18,19^ Previous research found no significant difference between the kinematic design of the files or the tip diameter and the amount of microorganisms extruded from the apical foramen.^20^ Furthermore, the effect of obturation materials and techniques on the quantity of AED has been studied several times.^7,10,21,22^ However, data regarding the effect of initial enlargement on AED during the removal of preceding filling materials are limited.

 Therefore, this study aimed to evaluate the AED during the removal of filling materials from teeth initially prepared up to ProTaper Universal (Dentsply Maillefer, Ballaigues, Switzerland) F3 or F4 files with or without the aid of a solvent (chloroform). The null hypothesis of this study was that neither solvent use nor the initial treatment would affect the quantity of AED.

## Methods

###  Tooth selection 

**Figure 1:**
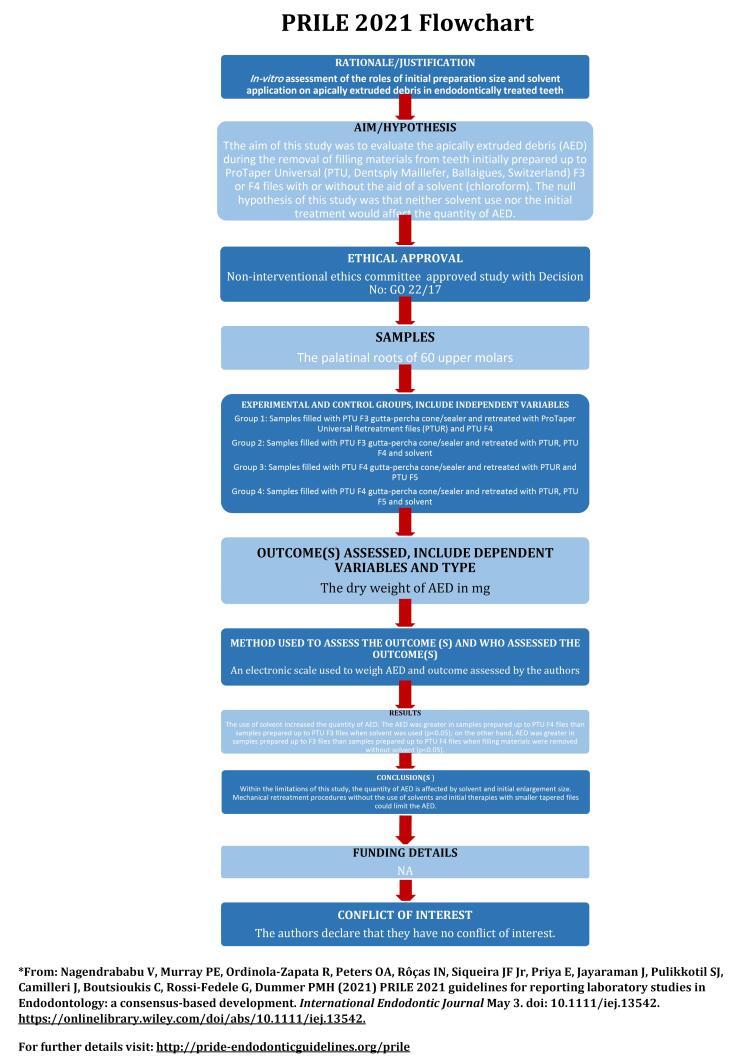
PRILE 2021 flowchart

 The manuscript of this laboratory study has been written according to Preferred Reporting Items for Laboratory Studies in Endodontology (PRILE) 2021 guidelines (Figure 1).^23^ Periodontally involved human maxillary molars were extracted and stored in sterile saline until use, following Ethics Committee approval (GO22/17). Considering a previous study,^10^ the sample size calculation was performed via software (G*Power ver. 3.1.9.4, Franz Faul, Universität Kiel, Germany), with an 80% power, a minimum of 13 samples per group required to show significant differences between the groups. The study included palatal roots with single roots and canals without root canal treatment, resorption, immature apex, caries, cracks, and fractures. In this research, sixty palatal roots of maxillary molars were used.

###  Initial therapy

 To standardize the length of the samples to 16.0 mm, a diamond disc mounted on a handpiece was used to decoronate them. The patency of palatal roots was determined using a #10 K (Dentsply Maillefer) file. The selected samples had straight roots and an initial apical diameter corresponding to #20 K-file. The working length (WL) was then determined using a #15 K file (Dentsply Maillefer) 1 mm shorter than the radiographic apex of the molars.

 According to the final file, the samples were randomly divided into two main groups: ProTaper Universal (PTU, Dentsply Maillefer, Ballaigues, Switzerland) F3 and ProTaper Universal F4. The samples prepared through the PTU F3 file were prepared using the PTU files in the following sequence: S1, SX, S2, F1, F2, and F3. This sequence was repeated in the PTU F4 group, and the F4 file was used additionally.

 The root canals were irrigated between each file with 2 mL of 2.5% sodium hypochlorite during mechanical shaping. Final irrigation was performed with 2 mL of 17% ethylenediamine tetraacetic acid. After drying the root canals with paper points, the samples were obturated with an epoxy resin-based sealer (AH Plus) and gutta-percha of the same dimension as the last file (F3 or F4 gutta-percha cone). The samples were radiographed to confirm the quality of the root canal obturation. The obturated samples were kept at 100% humidity and 37 °C for two weeks for the sealer to set.

###  Retreatment procedures

**Figure 2:**
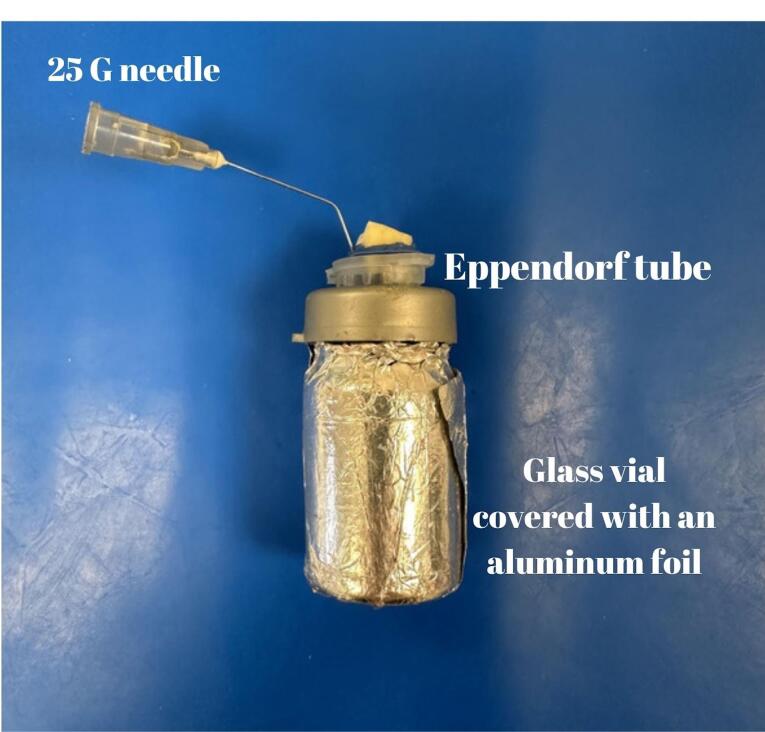
Experimental model system used to evaluate debris extrusion

 The debris was evaluated following Myers & Montgomery’s method.^24^ Briefly, the samples were placed in preweighed Eppendorf tubes via an electronic balance with an accuracy of 10^-4^ (Radwag, Radom, Poland). Holes were created in the caps of the tubes to insert samples into Eppendorf tubes. Using sticky wax (Polywax), the spaces between the samples and Eppendorf caps were sealed off. Eppendorf tubes with a cap-included sample were further inserted into a glass vial. A 25-G needle was placed near each sample before isolation to equalize air pressure inside and outside of the setup. To prevent the operator from observing debris extrusion during the instrumentation procedure, a glass vial was surrounded with aluminum foil, and a rubber dam sheet was used to cover the coronal part of the samples (Figure 2). Each main group was randomly divided into two subgroups: mechanical removal of the filling material with rotary files with or without a solvent (chloroform) (n = 15).

 The root canal filling materials were removed mechanically using ProTaper Universal Retreatment files (D1, D2, and D3, respectively) with a mild in-and-out motion to the WL, per the manufacturer’s instructions. The total solvent volume for each sample in the respective group was 0.04 mL of chloroform.^7^ Samples initially treated with PTU F3 were further treated with PTU F4 up to the WL, while the remaining samples were treated with PTU F5. Filling material removal was deemed complete according to previous studies,^7-11^ i.e., when no filling material could be seen on the instrument when WL could be reached, apical patency was confirmed, and the cleanliness of the root canal walls was visible. During removal procedures, 12 mL of distilled water was used for irrigation. Following the completion of removal procedures, the cap, syringe, and sample were removed from the tube, and the debris adhering to the root surface was collected by washing the root with 1 mL of distilled water in the tube. The tubes were stored for 14 days at 37 °C in an incubator to evaporate the distilled water. Each root canal was prepared by a single operator (Sİ). A second operator (EY) conducted weight calculations without knowledge of the group assignment. Using the previous weighing standards, the ultimate weight of the tubes, including the AED, was determined. The tubes were weighed three times, and the average value was calculated. To calculate the AED’s dry weight, the empty tube’s weight was subtracted from the weight of the tube containing the debris.

## Results

**Figure 3:**
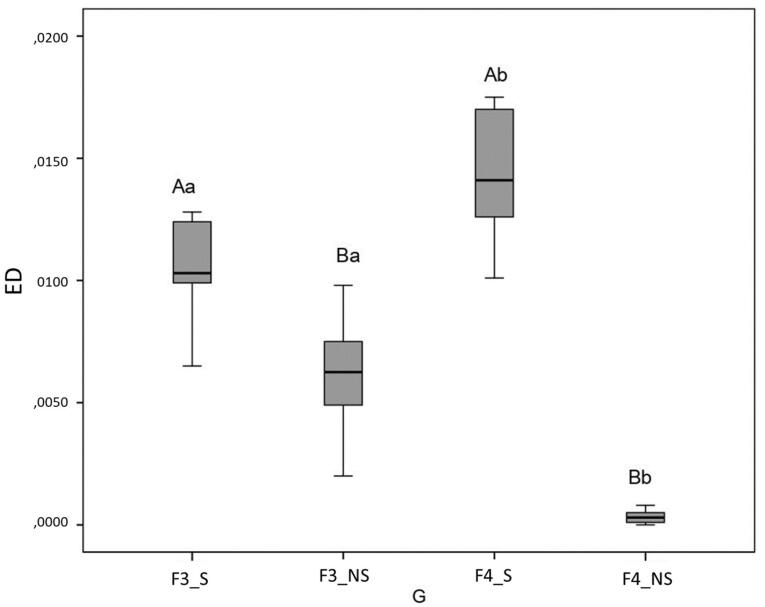
Box-plot graph of data representing the quantity of extruded debris of experimental groups in grams (A, B: revealed the statistical difference between samples initially prepared up to the same file and retreated with different methods. ED: Extruded Debris in gram, G: Groups, S: Solvent, NS: without solvent

 According to the Shapiro-Wilk test, data had a normal distribution. SPSS 20 was used to analyze data at *P* = 0.05 with two-way ANOVA and Bonferroni post hoc tests. Figure 3 displays the outcomes. The use of solvent increased the quantity of AED compared to samples retreated without solvent and initially prepared up to PTU F3 and F4 files (*P* < 0.05 for both comparisons). The AED was greater in samples initially prepared up to PTU F4 files than in samples prepared up to PTU F3 files when solvent was used (*P* < 0.05). On the other hand, AED was greater in samples initially prepared up to F3 files than in samples prepared up to PTU F4 files when filling materials were removed without a solvent (*P* < 0.05).

## Discussion

 AED can result in postoperative inflammation, exacerbations, and long-term failures.^12^ Therefore, various instrumentation techniques, obturation techniques, and obturation materials have been utilized to investigate the quantity of AED during filling material removal.^7-11,13,14,18-22^ All these variables affect AED. Manual instrumentation has been reported to increase the weight of AED compared to mechanical instrumentation.^13,14^ However, there is no consensus concerning the effect of parameters such as rotary file systems^4^ and solvent use^7-10^ on AED. Aldajani and Mathew^9^ reported that solvent use increased the weight of AED, whereas Çanakçi et al^7^ reported the opposite. In addition to these parameters, the effects of initial therapy parameters, such as obturation techniques^10,22^ and obturation materials^7,21^ on the AED were also evaluated. However, data on the effect of initial preparation size on the quantity of AED during retreatment procedures were limited. Therefore, this investigation evaluated AED during the mechanical or solvent-assisted (chloroform) removal of filling materials from teeth initially prepared up to PTU F3 or F4 and filled with PTU F3 or F4 gutta-percha cones in conjunction with a sealer, respectively.

 In the present study, chloroform was used as a solvent; it is toxic and should be used cautiously to avoid any complications during retreatment.^25,26^ As in the previous study,^7^ a limited amount of chloroform was used in the current study. According to the present study’s findings, the use of solvent increased the quantity of AED from samples filled with either PTU F3 or F4 gutta-percha cones because solvents soften the filling materials, and the pressure action of the files causes the softened and partially fluidized gutta-percha cones to move apically, not coronally. On the other hand, the elastic and rigid gutta-percha residues or fragments could occupy the grooves of the rotary files and move coronally in the absence of the solvent. One interesting result was that when the solvent was omitted, the quantity of AED from samples filled with F4 gutta-percha cones/sealers was lower than that of samples filled with F3 gutta-percha cones/sealers. In the present study, the average weight of 16-mm F4 gutta-percha cones was 19.80 mg, while the average weight of 16-mm F3 gutta-percha cones was 23.03 mg. This difference might be due to taper variations in the files, which could impact the weight of the AED. In contrast, when the solvent was used this time, samples filled with F3 gutta-percha cones/sealers extruded less debris than samples filled with F4 gutta-percha cones/sealers, possibly due to the ease of softening gutta-percha via solvents with small tapers as opposed to large ones. Furthermore, as the volume of gutta-percha decreases, the volume of sealer increases, which may impact the quantity of AED.

 The ProTaper multi-file system is one of the most commonly used endodontic systems.^27,28^ In the current study, only the PTU file system was utilized during both the initial treatment and retreatment phases. Compared to other file systems, such as ProTaper Next,^29^ ProTaper Universal Files have variable and large tapers. During initial preparations up to #40 with various file systems, the PTU system extruded more debris than ProTaper Next, WaveOne, or Reciproc files,^29^ and this finding was explained by file tapers, file numbers required to complete preparation, and cross-sectional design. PTU F3 or F4 was the final file used during the initial treatment in the current study. The diameters of the tips of PTU F3 and PTU F4 are 0.30 and 0.40 mm, and their taper in the first three millimeters is 0.09 and 0.06 mm, respectively. The last file used during retreatment was either PTU F4 or PTU F5. The tip diameter of PTU F5 is 0.50 mm, and its taper in the first three millimeters is 0.05 mm. According to a previous study,^20^ an increase in file diameter did not reveal a significant difference in AED; however, pressure and friction provided by the file could also affect the amount of AED.

 During extrusion experiments, primarily single-rooted premolar teeth were used.^8,9,11,13,19,20^ The mean minimum diameter of the apical foramina of maxillary palatal roots was greater than of mandibular premolar roots,^30^ and an increase in foramen diameter increases the risk of extrusion.^31^ In addition, maxillary molars have a complex root canal anatomy^32^ that reduces the success rate of initial treatment, and their roots may be located in the maxillary sinus, which increases the extrusion potential of filling materials, irrigants, and tissue debris.^33^ Therefore, palatal roots of maxillary molars were preferred in this study.

 The present study has numerous limitations. For instance, the *in vitro* design suspends the apex in air, whereas *in vivo*, it is surrounded by granulomatous or periradicular tissues, which may help to limit apical extrusion to a certain extent.^14,24^ There is no physical pressure exerted by the periapical tissues in vitro. Gravity may also play a role in the apical access and extrusion of irrigation solutions.^34^ Although the experimental setup does not fully reflect the clinic, it is the most commonly used in vitro comparison method. Moreover, it is challenging to identify the initial master apical file clinically during retreatment. In addition, distilled water was used for irrigation during retreatment, which is not consistent with clinical procedures. However, it has been reported that crystals formed after the evaporation of NaOCl could interfere with the quantity of AED.^35^ To prevent this effect of NaOCl, irrigation was performed with distilled water. Therefore, the findings of this study should be interpreted with caution.

## Conclusion

 Within the limitations of this study, the quantity of AED is affected by solvent use and initial enlargement size. Considering the potential for apical extrusion of filling materials, solvents should be used cautiously during root canal retreatment. Additionally, the taper of the files used during the initial preparation also affects the AED. Using files with smaller tapers, such as 0.04 and 0.06, may be beneficial when considering the amount of AED compared to those with larger tapers, such as 0.09.

## Competing Interests

 The authors declare that they have no conflict of interest.

## Ethical Approval

 Periodontally involved human maxillary molars were extracted and stored in sterile saline until use, following ethics committee approval (GO22/17).

## Funding

 Nil.
